# Precision Prevention of Recurrent Acute Pancreatitis: From Cause‐Oriented Diagnostics to Targeted Intervention

**DOI:** 10.1002/ueg2.70191

**Published:** 2026-02-19

**Authors:** Péter Hegyi

**Affiliations:** ^1^ Centre for Translational Medicine Semmelweis University Budapest Hungary; ^2^ Institute for Translational Medicine Medical School University of Pécs Pécs Hungary; ^3^ Institute of Pancreatic Diseases Semmelweis University Budapest Hungary; ^4^ Translational Pancreatology Research Group Interdisciplinary Centre of Excellence for Research Development and Innovation University of Szeged Szeged Hungary

Recurrent acute pancreatitis (RAP) remains one of the most clinically relevant yet under‐addressed trajectories in pancreatology. Up to 20%–30% of patients experience recurrent attacks after a first episode of acute pancreatitis (AP), and RAP is now clearly recognized as a major gateway toward early chronic pancreatitis (ECP), established chronic pancreatitis (CP), and long‐term metabolic sequelae, including diabetes [1]. Importantly, both structural pancreatic damage and endocrine dysfunction may evolve rapidly following recurrent episodes, particularly within the first years after the index attack [1]. The central clinical challenge is therefore no longer whether recurrence matters, but how precisely recurrence can be prevented.

Against this background, the randomized, double‐blind, placebo‐controlled trial by Cook et al., investigating naldemedine for the prevention of RAP, represents a conceptually important step forward [2]. Rather than applying a uniform preventive strategy, the study tests a mechanism‐driven intervention, targeting opioid‐related sphincter dysfunction and gut–pancreas signaling as a modifiable driver of recurrence. Although the trial was underpowered due to early termination, the direction and magnitude of the effect suggest a clinically meaningful signal. Importantly, the study is hypothesis‐generating rather than practice‐changing and challenges the field to move from generic recurrence prevention toward etiology‐ and mechanism‐specific strategies. However, precision prevention must begin upstream, with accurate identification of the underlying cause.

The strongest evidence for RAP prevention still lies in etiology identification and elimination. A post‐hoc analysis from the Dutch Pancreatitis Study Group demonstrated that in so‐called “presumed idiopathic” AP, extended diagnostic work‐up reveals an underlying cause in approximately one‐third of patients, most commonly occult biliary disease, and that treating the identified cause nearly halves recurrence rates [3]. Similarly, biliary pancreatitis, even in high‐risk settings such as pregnancy, shows dramatically reduced recurrence when definitive source control is achieved. The multinational BORN study confirmed that timely cholecystectomy or ERCP is both safe and effective, with near‐elimination of recurrent attacks [4]. Recent reviews further emphasize that failure to apply systematic EUS‐ and MRCP‐based diagnostic algorithms perpetuates avoidable RAP [5]. Together, these data firmly establish that precision prevention begins with diagnostic rigor, not pharmacology.

Alcohol‐induced RAP remains the most frequent, however the most preventable form of recurrence. A structured, in‐hospital brief intervention program has been shown to significantly reduce alcohol consumption and biochemical relapse markers, with clear implications for recurrence prevention [6, 7]. This represents a form of behavioral precision prevention, in which the dominant driver is neither ductal nor inflammatory, but a modifiable lifestyle exposure that can be addressed through targeted intervention.

Why does preventing recurrence matter so much? Because recurrence drives progression. Experimental, cross‐sectional, and longitudinal human data converge on a critical threshold: three or more acute pancreatitis episodes. Beyond this point, molecular, morphologic, and clinical features become indistinguishable from early chronic pancreatitis [8]. The Goulash‐Plus cohort further demonstrates that pancreatic structural damage and endocrine dysfunction accelerate most rapidly during the early years following recurrent attacks [1]. From a prevention standpoint, RAP should therefore be viewed as a time‐sensitive intervention window, rather than a series of isolated acute events [9].

In selected patients with ductal pathology, such as stones, strictures, or ductal hypertension, endoscopic precision is also essential. Long‐term data on digital single‐operator pancreatoscopy‐guided lithotripsy demonstrate sustained symptom control and reduced inflammatory flares when complete ductal clearance is achieved [10]. Although these interventions have primarily been studied in established CP, they are highly relevant to RAP patients at risk of structural progression, where timely ductal decompression may alter the disease trajectory.

In this broader context, the trial by Cook et al. should not be interpreted as a universal preventive solution, but rather as a prototype of precision pharmacologic prevention [2]. Its true contribution lies in demonstrating that RAP prevention can, and should, be biologically stratified. Future studies must define which RAP phenotypes derive the greatest benefit from such interventions and how pharmacologic strategies can be integrated with diagnostic, behavioral, and interventional approaches.

In conclusion, the era of “one‐size‐fits‐all” prevention in recurrent acute pancreatitis is drawing to a close. The accumulated evidence consistently points toward precision prevention: exhaustive etiologic work‐up, strict guideline adherence, targeted lifestyle and endoscopic interventions, and mechanism‐based pharmacologic strategies. The study by Cook et al. does not provide the final answer, but it redefines the question, and that may represent the most important step toward truly preventing recurrent acute pancreatitis.

## Conflicts of Interest

The author declares no conflicts of interest.

## Data Availability

Data sharing not applicable to this article as no datasets were generated or analyzed during the current study.

## References

[1] A. Mikó , N. Farkas , Á. Vincze , et al., “Progression of Pancreatic Morphologic Changes and Endocrine Dysfunction After Acute Pancreatitis: Preliminary Results of the Longitudinal Goulash‐Plus Cohort Study,” Gastroenterology (2026), 10.1053/j.gastro.2025.09.034. 41556859

[2] M. E. Cook , C. S. Knoph , L. Davidsen , et al., “Naldemedine for the Prevention of Recurrent Acute Pancreatitis: A Randomised, Double‐Blind, Placebo‐Controlled Trial,” United European Gastroenterology Journal 14, no. 1 (2026): e70178, 10.1002/ueg2.70178. 41553774 PMC12815247

[3] N. D. Hallensleben , D. S. Umans , S. A. Bouwense , et al., “The Diagnostic Work‐Up and Outcomes of 'Presumed' Idiopathic Acute Pancreatitis: A Post‐Hoc Analysis of a Multicentre Observational Cohort,” United European Gastroenterology Journal 8, no. 3 (2020): 340–350, 10.1177/2050640619890462. 32213015 PMC7184667

[4] D. Tarján , E. Szalai , B. Erőss , et al., “Safety and Effectiveness of Cholecystectomy and Endoscopic Retrograde Cholangiopancreatography in Biliary Pancreatitis During Pregnancy: BORN Study,” United European Gastroenterology Journal 13, no. 9 (2025): 1803–1811, 10.1002/ueg2.70121. 41045491 PMC12606018

[5] A. Aronen , L. Guilabert , A. Hadi , et al., “Idiopathic Acute Pancreatitis (IAP)‐a Review of the Literature and Algorithm Proposed for the Diagnostic Work‐up of IAP,” Translational Gastroenterology and Hepatology 9 (2024): 71, 10.21037/tgh-23-125. 39503029 PMC11535791

[6] R. Nagy , K. Ocskay , A. Váradi , et al., “In‐Hospital Patient Education Markedly Reduces Alcohol Consumption After Alcohol‐Induced Acute Pancreatitis,” Nutrients 14, no. 10 (2022): 2131, 10.3390/nu14102131. 35631272 PMC9144493

[7] I. Nordback , H. Pelli , R. Lappalainen‐Lehto , S. Järvinen , S. Räty , and J. Sand , “The Recurrence of Acute Alcohol‐Associated Pancreatitis Can Be Reduced: A Randomized Controlled Trial,” Gastroenterology 136, no. 3 (2009): 848–855, 10.1053/j.gastro.2008.11.044. 19162029

[8] P. J. Hegyi , A. Soós , E. Tóth , et al., “Evidence for Diagnosis of Early Chronic Pancreatitis After Three Episodes of Acute Pancreatitis: A Cross‐Sectional Multicentre International Study With Experimental Animal Model,” Scientific Reports 11, no. 1 (2021): 1367, 10.1038/s41598-020-80532-6. 33446814 PMC7809468

[9] M. S. Petrov and D. Yadav , “Global Epidemiology and Holistic Prevention of Pancreatitis,” Nature Reviews Gastroenterology & Hepatology 16, no. 3 (2019): 175–184, 10.1038/s41575-018-0087-5. 30482911 PMC6597260

[10] C. C. Conrad , M. Ellrichmann , M. Bronswijk , et al., “Long‐Term Efficacy and Safety of Digital‐Single‐Operator‐Video‐Pancreatoscopy Guided Lithotripsy for Pancreatic Duct Stones,” United European Gastroenterology Journal 13, no. 7 (2025): 1127–1132, 10.1002/ueg2.70063. 40478689 PMC12463695

